# A Temperature-Sensitive Polymeric Rheology Modifier Used in Water-Based Drilling Fluid for Deepwater Drilling

**DOI:** 10.3390/gels8060338

**Published:** 2022-05-30

**Authors:** Zhongyi Wang, Jinsheng Sun, Kun Zhang, Kaihe Lv, Xianbin Huang, Jintang Wang, Ren Wang, Xu Meng

**Affiliations:** 1 Key Laboratory of Unconventional Oil & Gas Development, China University of Petroleum (East China), Ministry of Education, Qingdao 266580, China; wzy0512xx@126.com (Z.W.); lkh54321@126.com (K.L.); 20170092@upc.edu.cn (X.H.); wangjintang163@126.com (J.W.); s21020004@s.upc.edu.cn (X.M.); 2 School of Petroleum Engineering, China University of Petroleum (East China), Qingdao 266580, China; 3 CNPC Bohai Drilling Engineering Company Limited, Tianjin 300270, China; zhangkunzk@cnpc.com.cn; 4 CNPC Engineering Technology R & D Company Limited, Beijing 102206, China; wangrdr@cnpc.com.cn

**Keywords:** temperature-sensitive, rheology modifier, water-based drilling fluid, deepwater drilling

## Abstract

Rheology modifiers are essential for the flat rheology of water-based drilling fluids in deepwater. The low temperature thickening of deepwater water-based drilling fluids results in dramatic rheological changes in the 20–30 °C range. To address such problems, NIPAM with a self-polymerized product LCST of 32–35 °C was selected as the main body for synthesis. While introducing the hydrophilic monomer AM to enhance the thickening properties, the hydrophobic monomer BA was selected to reduce the LCST of the product. In this paper, a temperature-sensitive polymeric rheology modifier (PNBAM) was synthesized by emulsion polymerization using *N*-isopropyl acrylamide, acrylamide, and butyl acrylate as monomers. The PNBAM was characterized using infrared spectroscopy (FT-IR), thermogravimetric analysis (TGA), and nuclear magnetic resonance hydrogen spectroscopy (NMR). The rheological properties, temperature resistance, and salt resistance of PNBAM in the base fluid (BF) were tested. The performance of PNBAM in the drilling fluid system was also evaluated, and a water-based drilling fluid system of flat rheology for deepwater was formulated. The rheological modification mechanism of PNBAM was analyzed by turbidity analysis, particle size analysis, and zeta analysis. Experimental results show that PNBAM has good rheological properties. PNBAM is temperature resistant to 150 °C, salt-resistant to 30 wt%, and calcium resistant to 1.0 wt%. PNBAM also has good flat rheology characteristics in drilling fluid systems: AV_4°C_:AV_25°C_ = 1.27, PV_4°C_:PV_25°C_ = 1.19. Mechanistic analysis showed that the LCST (Lower Critical Solution Temperature) of 0.2 wt% PNBAM in an aqueous solution was 31 °C. Through changes in hydrogen bonding forces with water, PNBAM can regulate its hydrophilic and hydrophobic properties before and after LCST, which thus assists BF to achieve a flat rheological effect. In summary, the temperature-sensitive effect of PNBAM has the property of enhancing with increasing temperature. While the tackifying effect of conventional rheology modifiers diminishes with increasing temperature, the temperature-sensitive effect of PNBAM gives it an enhanced thickening effect with increasing temperature, making it a more novel rheology modifier compared to conventional treatment additives. After LCST, compared to conventional rheology modifiers (XC), PNBAM has a more pronounced thermo-thickening effect, improving the main rheological parameters of BF by more than 100% or even up to 200% (XC less than 50%). This contributes to the flat rheology of drilling fluids. PNBAM has good application prospects and serves as a good reference for the development of other rheology modifiers.

## 1. Introduction

The world’s marine oil resources account for 34% of the world’s total oil resources, of which the proven reserves are about 38 billion tons [1,2,3,4,5,6,7,8,9,10]. With oil reserves on land and in shallow seas decreasing year by year, the development of deep-water oil and gas resources has received attention. Many countries and regions around the world are actively pursuing research into offshore oil and gas drilling technology, with the most active areas including the Gulf of Mexico, West Africa, Norway, and Brazil [11,12]. This has led to a steady rise in offshore oil and gas production and a gradual shift in oil and gas drilling operations from shallow coastal waters to deeper waters.

The South China Sea is rich in oil and gas resources, accounting for about one-third of China’s total oil and gas resources [13,14]. After more than 20 years of oil and gas exploration and development in the South China Sea, nearly 1 billion tons of oil resources and more than 300 billion cubic meters of natural gas reserves have been surveyed in the northern shelf area of the South China Sea, with an accumulated oil production of up to 12 million tons and more than 3 billion cubic meters of gas production [15,16].

These oil and gas resources are only produced from the shelf area, with more than 60% of the oil and gas reserves buried in deepwater areas that need to be explored and developed [17]. In recent years, China has made certain achievements in the exploration and development of petroleum geology in shallow waters; however, the exploration of deepwater areas is still in its initial stage. With the continuous strengthening of offshore oil exploration and development and the increasing expansion of the development area, China’s offshore drilling operations will continue to march into deep water areas.

As deepwater drilling continues to develop, the complex environment of deepwater poses many complications for deepwater drilling compared to conventional drilling. One of the most challenging issues posed is the rheology of drilling fluids due to the low temperature conditions in deepwater drilling [18,19,20,21,22]. It is not uncommon to see mud line temperatures of 2–4 °C in deepwater environments [18,19,20,21,22]. Low temperatures can cause a significant increase in the viscosity and gel strength of drilling fluids and even significant gelling in drilling fluids, causing problems with high equivalent circulating density [18,19,20,21,22].

In deepwater drilling operations, there are high subsea hydrostatic column pressures and low shallow compaction. These conditions allow for a narrow window of pore pressure and fracture pressure in the deepwater surface layer, which in turn leads to problems with the high equivalent circulating density of drilling fluid [18,19,20,21,22]. These problems can easily lead to major incidents, such as formation fractures, loss of solution at the casing shoe, and more serious well leaks. The concept of “flat rheology” has been developed for deepwater drilling fluid applications to address such problems [20,23,24,25,26].

This concept, which aims to effectively control equivalent circulating density (ECD) and reduce drilling fluid losses, refers to maintaining a relatively constant apparent viscosity, plastic viscosity, yield value, and θ6, θ3 readings over a temperature range of 4–65 °C [23,24,25,26]. The definition of ‘flat rheology’ is mostly determined by measuring the ratio of the apparent viscosity, plastic viscosity, and yield value of the drilling fluid at different temperature points [23,24,25,26]. Temperature points are usually chosen from 4, 25, and 65 °C.

After being introduced in deepwater drilling fluids, the definition of flat rheology was first widely used and worked well in synthetic and oil-based drilling fluids. In dispersion systems, the influence of the dispersion medium on the viscosity of the system is significant. The base oils commonly used in oil-based and synthetic-based drilling fluids are strongly influenced by temperature variations [23,24,25,26]. To address such problems, scholars have developed new mineral oils or biodiesels with low temperature and low viscosity properties as base oils [27,28].

The effect of different drilling fluid additives on the constant rheological performance of synthetic-based drilling fluid systems from 4–65 °C is also analyzed, and new emulsifiers, rheology modifiers, and viscosity builders are selected or developed to formulate drilling fluid additives that can reduce key viscosity parameters at low temperatures and increase these parameters at high temperatures [20]. From these two aspects, synthetic and oil-based drilling fluids with flat rheological properties will be developed.

Flat rheology drilling fluids, although widely and effectively used in synthetic and oil-based drilling fluids, are limited in their application due to the cost and environmental contamination issues [29,30]. However, water-based drilling fluids offer better environmental performance and lower costs compared with synthetic and oil-based fluids. The viscosity of clay in water-based drilling fluids has the characteristic of decreasing with temperature and increasing with decreasing temperature [21].

Similarly, the viscosity of the polymer in water-based drilling fluids varies with temperature in the same way as clay [21]. This situation is not conducive to the low temperature rheological regulation of water-based drilling fluids during deepwater drilling. Currently, polyamine drilling fluids are widely used in deepwater drilling. However, studies have shown that the rheological properties of polyamine fluids also vary significantly over a wide range of temperatures. Water-based drilling fluids, such as polyamines, still have problems with ECD control and leakage [31].

A ZnTiO_3_ nanoparticle was developed by Maryam Edalatfar et al. [32]. It was found that the addition of 1 wt% of ZnTiO_3_ nanoparticles, even in the presence of 2% and 4% salt, at 25 and at 90 °C, respectively. it was able to maintain the shear thinning behavior of mud-containing nanoparticles well at different shear rates. However, the level rheological properties were not explored in more depth. Some scholars have reduced the amount of bentonite added or developed solid-free water-based drilling fluids to give better rheological properties to water-based drilling fluids.

An amphoteric polymer P(AM-DMC-AMPS) was developed by Xin Zhao et al. [33]. WBDF containing P(AM-DMC-AMPS) has lower and more stable rheological properties due to its shorter molecular chains. It also improves the filtration performance of WBDF and is compatible with other WBDF components. However, the lack of bentonite can lead to severe barite sagging and settling. Some scholars have addressed the problem of water-based drilling fluids with rheological properties that vary greatly over a wide temperature range by developing thermosensitive polymers.

Binqiang Xie et al. [22]. developed a new thermosensitive copolymer (PANA) and found that the rheology of PANA-based fluids was more stable than that of typical WBDF. The main rheological parameters, such as the apparent viscosity (AV), yield value (YP), and low shear rate viscosity (LSRV), of PANA fluids varied by less than 15%. However, their performance has not been evaluated in drilling fluid systems.

According to the above problem, a temperature-sensitive polymeric rheology modifier PNBAM for deepwater drilling water-based drilling fluids was synthesized using acrylamide (AM), butyl acrylate (BA), and N-isopropyl acrylamide (NIPAM) as monomers in a free radical emulsion polymerization reaction. In this polymer, the isopropyl acrylamide side chain acts as the thermosensitive group, and the amide group and the butyl ester side chain act as the hydrophilic and hydrophobic side chains. In particular, the temperature-sensitive side chains provide a temperature-sensitive effect for PNBAM, resulting in a tackifying effect that differs from that of conventional rheology modifiers with increasing temperature.

The hydrophilic side chains further enhance the tackifying effect of PNBAM. However, as the introduction of hydrophilic side chains enhances the LCST of PNBAM, hydrophobic side chains are introduced to further reduce the LCST of the product. At the same time, the adsorption of amide groups on bentonite particles further enhances the effect of PNBAM in BF. With increasing temperature, PNBAM has a good effect on tackifying effect and increasing gel strength in bentonite-based slurries. The drilling fluid system using PNBAM as the key material has good constant rheological properties in the range of 4–65 °C. The flat rheology mechanism of PNBAM in water-based drilling fluids was also comprehensively studied.

## 2. Results and Discussion

### 2.1. Characterization of PNBAM

#### 2.1.1. FT-IR

**Figure 1:**
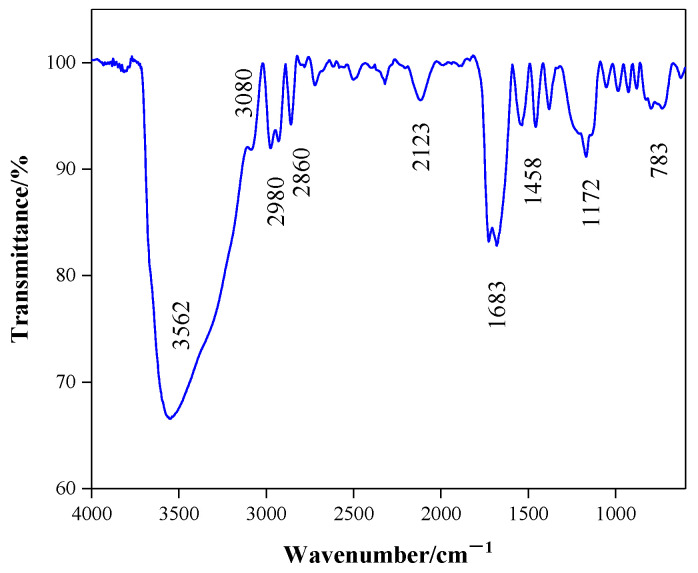
FT−IR of PNBAM.

Figure 1 shows the FT-IR of PNBAM. As shown in Figure 1, the stretching vibrational peaks of the N–H, C=O, and C-N bonds present in the amide group occur at 3562, 1683, and 1172 cm^−1^, respectively [34,35,36]. The stretching vibration peaks of –CH_3_ and –CH_2_ in the isopropyl group appear at 3080 and 2980 cm^−1^, respectively, and the bending vibration peak appears at 1458 cm^−1^ [36,37]. The stretching vibration peak of the C–O bond present in the ester bond occurs at 783 cm^−1^ [36,38]. The FT–IR results show that PNBAM contains functional groups characteristic of the various monomers.

#### 2.1.2. H-NMR Spectra

**Figure 2:**
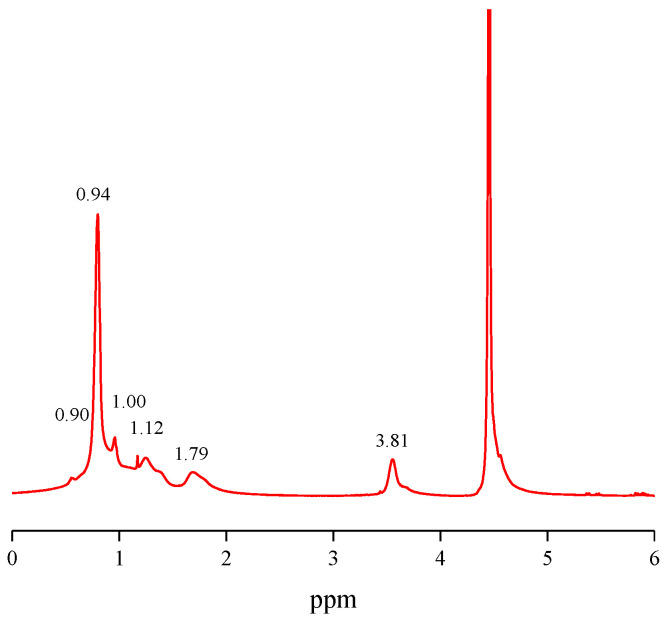
NMR for PNBAM.

Figure 2 shows the NMR hydrogen spectrum of PNBAM. As shown in Figure 2, the absence of absorption peaks above chemical shift 5 in the NMR spectrum indicates that no olefins are present, indicating that the reaction is sufficient and that no monomers are present that do not participate in the reaction. The triple peaks at 0.90, 0.94, 1.00, and 1.12 ppm are characteristic peaks for methyl groups from the butane side chain and isopropyl groups [33,37]. The characteristic peak for methylene is at 1.79 ppm [33,37]. The characteristic peak at 3.81 ppm is the C-H single bond brought about by the amino group and the isopropyl group [33,37]. In summary, the molecular structure of PNBAM was further analyzed by NMR, and the synthesized product PNBAM conforms to the design.

#### 2.1.3. TGA

**Figure 3:**
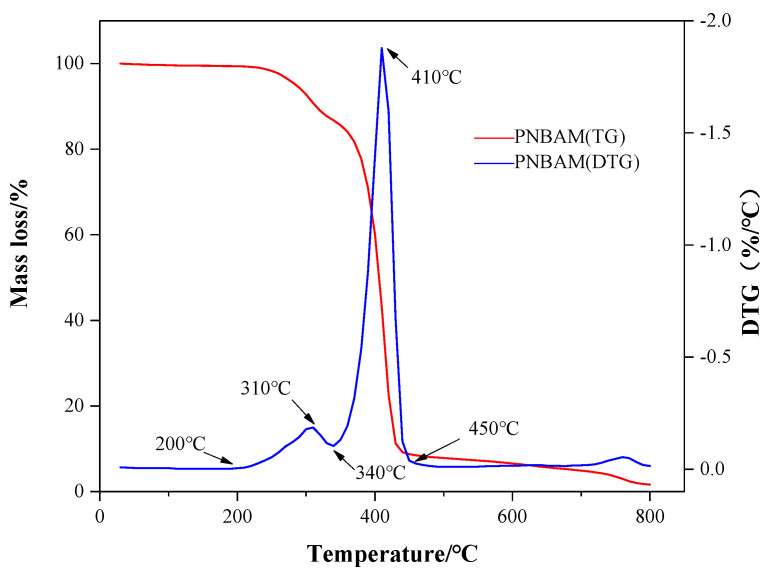
TG/DTG for PNBAM.

Figure 3 shows the TG/DTG of PNBAM. As shown in Figure 3, the thermal decomposition process of PNBAM can be divided into three stages. The first stage occurred between 30 °C and 200 °C. At this stage, the weight loss of the sample was 0.61%. PNBAM contains hydrophilic groups, resulting in a certain amount of adsorbed and bound water in the outer layer of the molecule, which is first evaporated during the warming process [36,37,39]. The second stage occurred between 200 and 340 °C. This stage decomposed fastest at 310 °C. At this stage, part of the PNBAM structure decomposed. According to the literature analysis, the side chain structure decomposed at this stage is believed to be a butyl ester side chain [40,41].

The third stage occurred between 340 and 450 °C, where PNBAM started to decompose heavily and rapidly around 340 °C, reaching the fastest decomposition rate at 410 °C. This phenomenon indicated that the internal structure of the polymer molecule has been damaged and that the C–C backbone of PNBAM started to decompose [36,37,39]. In summary, the initial thermal decomposition temperature of PNBAM was 200 °C, and the weight loss at 310 °C was only 9.23%, indicating the high structural stability and good thermal stability of PNBAM.

### 2.2. Performance of PNBAM

#### 2.2.1. Rheological Performance of PNBAM

**Figure 4:**
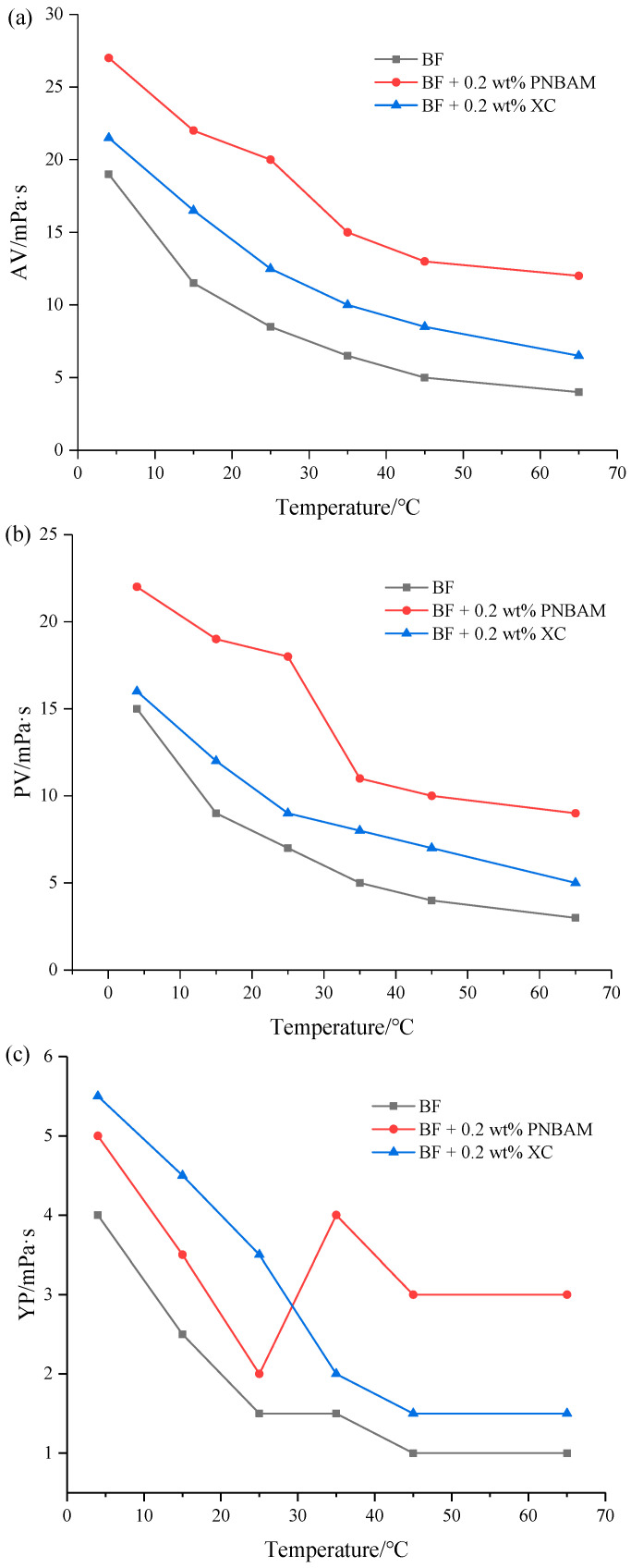
Effects of XC and PNBAM on AV (**a**), PV (**b**), YP (**c**) of BF after aging at 120 °C for 16 h where AV is the apparent viscosity (mPa·s), PV is the plastic viscosity (mPa·s), and YP is the yield value (Pa).

**Figure 5:**
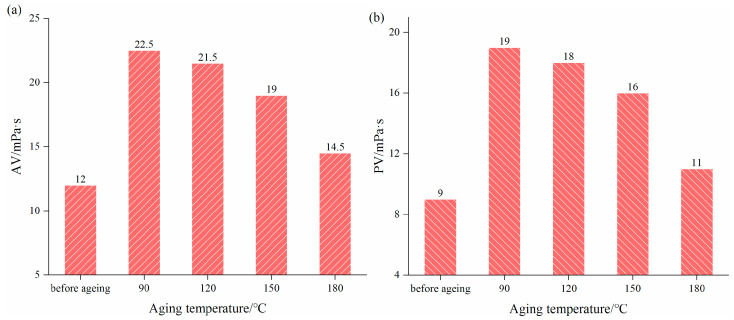

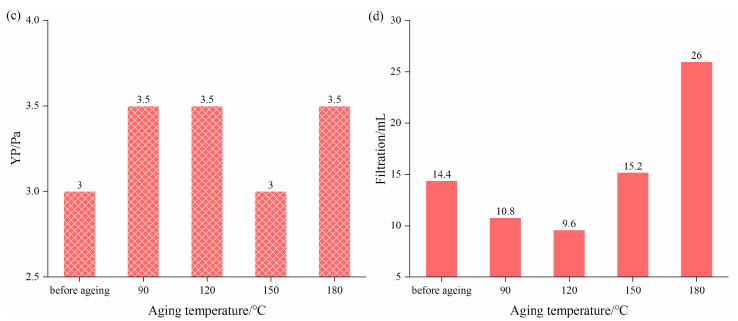
Effect of ageing temperature on the AV (**a**), PV (**b**), YP (**c**), and filtration (**d**) performance of PNBAM in BF.

Figure 4 and Table 1 show the rheological curves and parameter ratios of different BFs with temperature. As can be seen from Figure 5 and Table 2, in BF: AV_4__°C_:AV_25°C_ = 2.23, AV_4°C_:AV_65°C_ = 4.75; in BF + 0.2 wt% XC: AV_4__°C_:AV_25°C_ = 1.72, AV_4__°C_:AV_65°C_ = 3.31, in BF + 0.2 wt% PNABAM: AV_4__°C_:AV_25°C_ = 1.35, AV_4__°C_:AV_65°C_ = 2.25. Both additives have a certain tackifying effect at low temperatures, with XC having a weaker tackifying side effect at low temperatures.

The amide group of PNBAM adsorbs to the clay at low temperatures, leading to an increase in low temperature viscosity [21,22,42]. The multi-hydroxyl group of XC interacts with water molecules to form a three-dimensional hydrated network structure, which has a much weaker effect on the clay [21]. As the temperature rises, the viscosity of BF decreases, at which point PNBAM has a stronger tackifying effect on BF than XC, reaching a stronger tackifying effect after LCST and a higher AV lift of up to 200% on the base slurry (50% for XC).

As can be seen from Figure 4 and Table 1, In BF: PV_4__°C_:PV_25__°C_ = 2.14, PV_4__°C_:PV_65__°C_ = 5.00; in BF + 0.2 wt% XC: PV_4__°C_:PV_25__°C_ = 1.77, PV_4__°C_:PV_65__°C_ = 3.20; in BF + 0.2 wt% PNABAM: PV_4__°C_:PV_25__°C_ = 1.22, PV_4__°C_:AV_65__°C_ = 2.44. In terms of regulating the PV of the BF of the two drilling fluid additives, XC has a weaker tackifying side effect at low temperatures (4 °C).

PNBAM still shows better performance as the temperature increases, with a PV increase of >100% (XC less than 50%). Analysis of the molecular thermodynamic motion and the associated drilling fluid rheology suggests that both drilling fluid additives inevitably have a negative effect of tackifying effect at low temperatures. The ratio of yield value parameters for BF is YP_4°C_:YP_65°C_ = 4.00, for BF + 0.2 wt% PNBAM is YP_4°C_:YP_65°C_ = 1.67, and for BF + 0.2 wt% XC is YP_4°C_:YP_65°C_ = 3.67. As the temperature increases, PNBAM has a significantly better tackifying effect than XC, and PNBAM still has an increase in tackifying effect after LCST.

#### 2.2.2. Temperature Resistance Analysis

**Figure 6:**
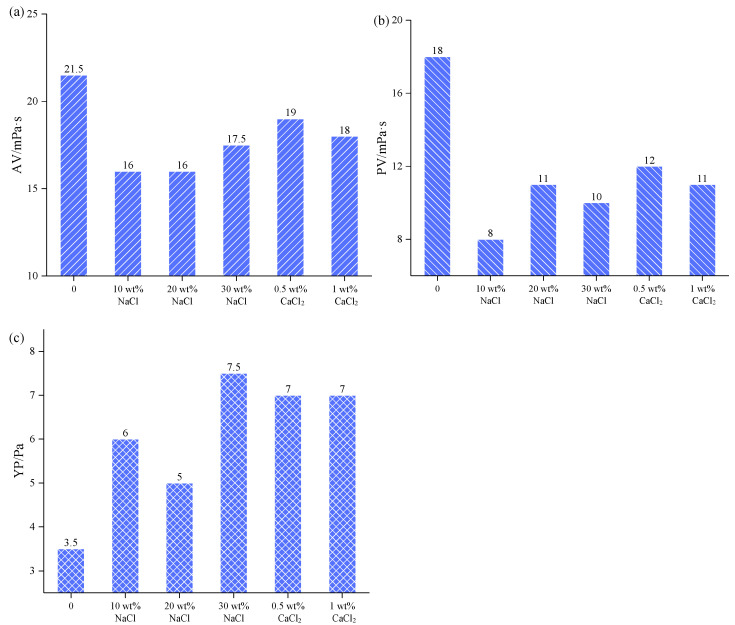
Effects of salt concentration on the AV (**a**), PV (**b**), and YP (**c**) performance of PNBAM in BF.

Figure 5 shows the performance of AV, PV, YP, and API filtration for BF + 0.2 wt% PNBAM after ageing at different temperatures. As can be seen from Figure 6, AV changes from 22.5 to 14.5 mPa·s at an ageing temperature of 90 to 180 °C, PV changes from 19 to 11 mPa·s at an ageing temperature of 90 to 180 °C, YP changes from 3.5 to 3 Pa at an ageing temperature of 90 to 150 °C. With increasing ageing temperature, the values of AV, PV, and YP parameters of the aged BF with the addition of PNBAM gradually decrease. However, all are higher than the pre-ageing.

The API filtration of BF + 0.2 wt% PNBAM after different aging was 10.8 mL (90 °C), 9.6 mL (120 °C), 15.2 mL (150 °C), and 26 mL (180 °C), while the API filtration before ageing was 14.4 mL. The API filtration of the BF after ageing at 90 °C, 120 °C, and 150 °C decreased or increased slightly compared to before ageing. However, after ageing at 180 °C, the API filtration of the BF showed a substantial increase.

The reasons for the reduction of API filtration are the amide groups in the PNBAM molecular chain can be adsorbed on the clay surface to form a dense film structure [37,39]. This situation can effectively improve the quality of the mud cake and reduce API filtration [37,39]. At the same time, after the introduction of hydrophobic groups, PNBAM still has hydrophobic links between the molecular chains before its LCST.

The molecular chains are entangled with each other and form a network structure, which has a better effect of tackifying effect and reduces API filtration [37,39]. The viscosity of the BF after ageing at 180 °C is essentially similar to that before ageing, and the API filtration after ageing is substantially higher at this point, indicating complete polymer breakdown failure at this point. After aging at 150 °C, the BF with PNBAM has suitable viscosity and API filtration, indicating that PNBAM is resistant to high temperatures at 150 °C.

#### 2.2.3. Salt and Calcium Resistance Analysis

**Figure 7:**
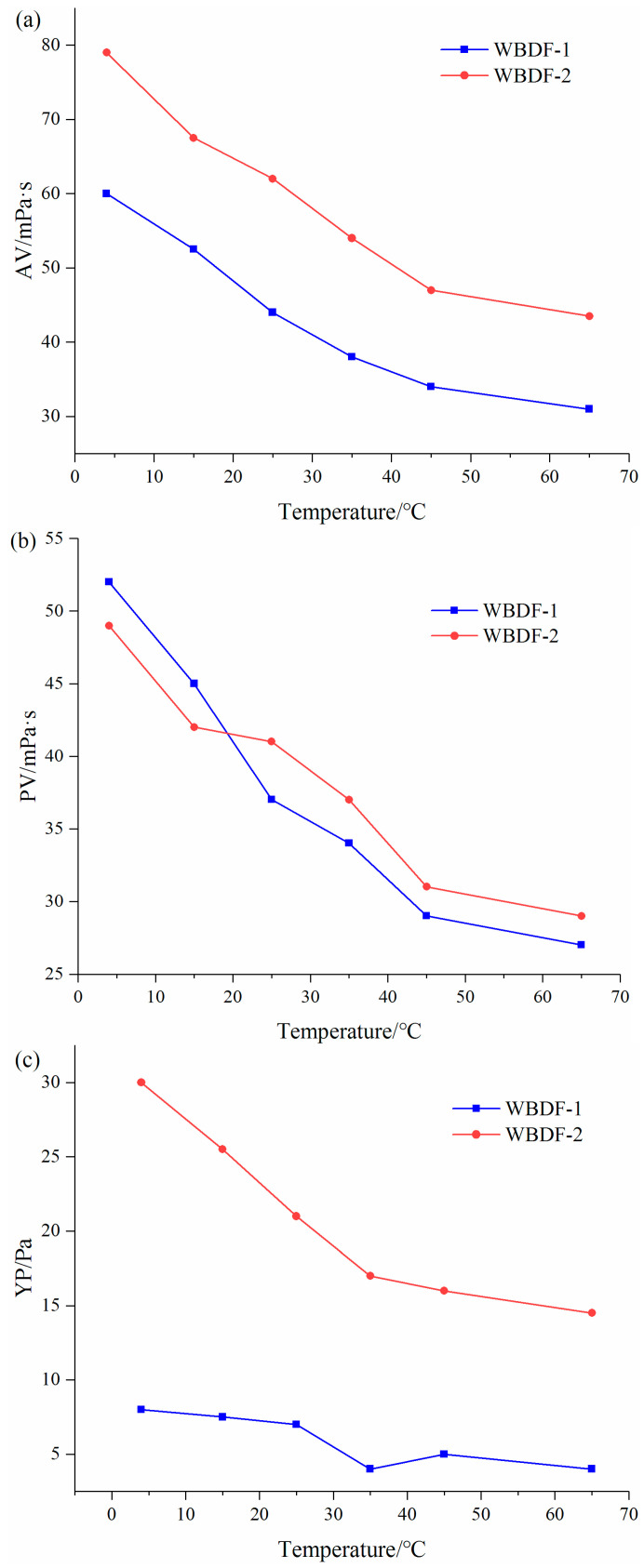
Effect of PNBAM on the AV (**a**), PV (**b**), and YP (**c**) performance of WBDF (water-based drilling fluids).

Figure 6 shows the variation of the AV, PV, and YP with salt addition of BF + 0.2 wt% PNBAM. As can be seen from Figure 7, after ageing at 120 °C for 16 h, the BF containing 0.2 wt% PNBAM first showed a small decrease in the AV, PV, and YP as the salt addition increased. The presence of NaCl compresses the diffuse double layer of the bentonite particles and reduces the dispersion of the bentonite particles. On the other hand, when the salt addition is not high (10 wt% NaCl), there is a “face-to-face” association of the clay particles, which reduces the dispersion of the clay and causes a decrease in the AV, PV, and YP of the BF [43].

However, as the salt concentration continues to increase (20 wt% NaCl and 30 wt% NaCl), the viscosity of the BF with PNBAM increases slightly. This due to the addition of salt, which enhances the polarity of the water, which reduces the LCST of PNBAM to the ambient temperature range (20–30 °C) or even lower, resulting in a temperature-sensitive effect and a certain tackifying effect. Similarly, after CaCl_2_ addition (0.5 wt% CaCl_2_), the rheological parameters of the BF decreased to a certain extent. However, with the increase of CaCl_2_ addition (1.0 wt% CaCl_2_) the rheological parameters of the BF did not show any significant changes. This indicates that PNBAM has good salt and calcium resistance.

According to Table 1, the drilling fluid system has good conventional performance, a moderate range of rheological parameters, and low filtration. As can be seen from Figure 7, the addition of the rheology modifier PNBAM increased the values of AV, PV, and YP of the drilling fluid from 4 to 65 °C to an extent. As the temperature increased, the rate of enhancement of each rheological parameter of the drilling fluid gradually increased.

#### 2.2.4. Performance of Drilling Fluid System

As can be seen from Table 3, compared to WBDF-1, the WBDF-2 has a certain degree of reduction in the rheological parameter ratios of both AV and PV, which plays a certain effect on the low-temperature rheology regulation.

In previous studies, deepwater water-based drilling fluid systems with AV_4°C_:AV_25°C_ < 1.5 and PV_4°C_:PV_25°C_ < 1.50 were considered to have a certain level of rheological performance. The WBDF-2 with AV_4°C_:AV_25°C_ < 1.30 and PV_4°C_:PV_25°C_ < 1.20 indicates that the preferred drilling fluid system has a good performance in flat rheology [33].

### 2.3. Mechanism Study

#### 2.3.1. Influencing Factors of LCST

**Figure 8:**
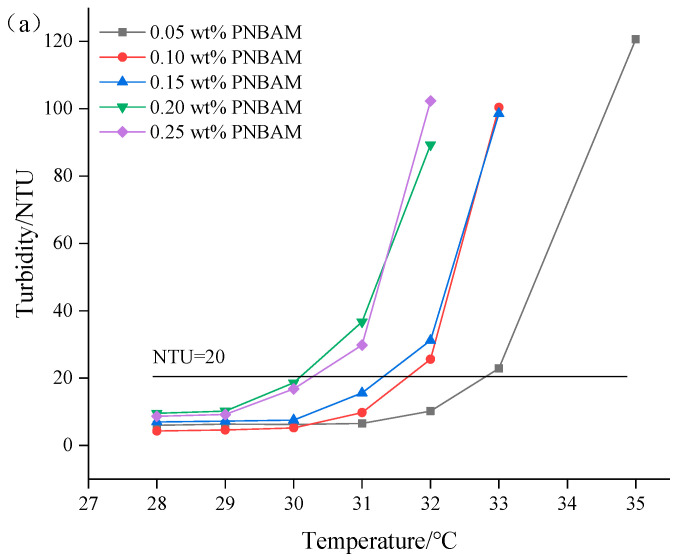

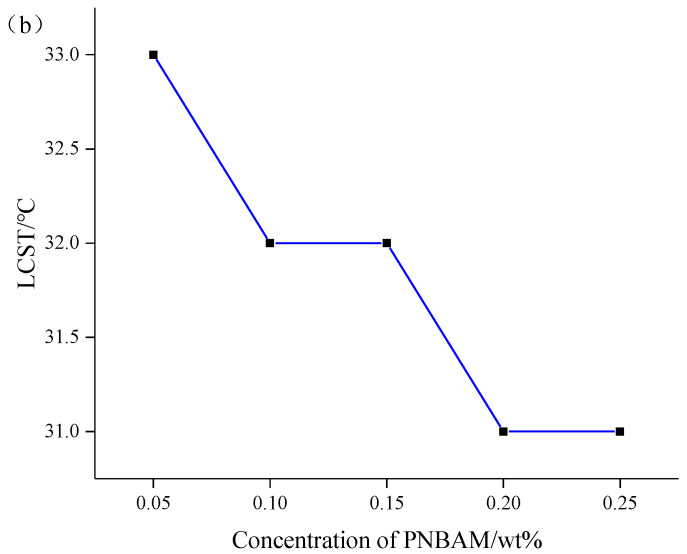
Effects of the concentration (wt%) on turbidity change with temperature (**a**) and LCST (**b**) of PNBAM aqueous solution.

Figure 8 shows the variation of LCST with the concentration of PNBAM. As can be seen from Figure 8, with the concentration of PNBAM increasing, its LCST gradually decreased; however, the decrease in LCST of PNBAM is not high. The rising addition enhances the number of hydrogen bonds bound between PNBAM and water. However, it has little effect on the polarity of the water itself and thus has a weak effect on the strength of the hydrogen bonds. This indicated that the addition of quantity has a weak effect on the LCST of PNBAM. LCST at 31 °C for both 0.25 wt% and 0.20 wt% addition of PNBAM. Therefore, 0.20 wt% was chosen as the optimum dose for PNBAM.

**Figure 9:**
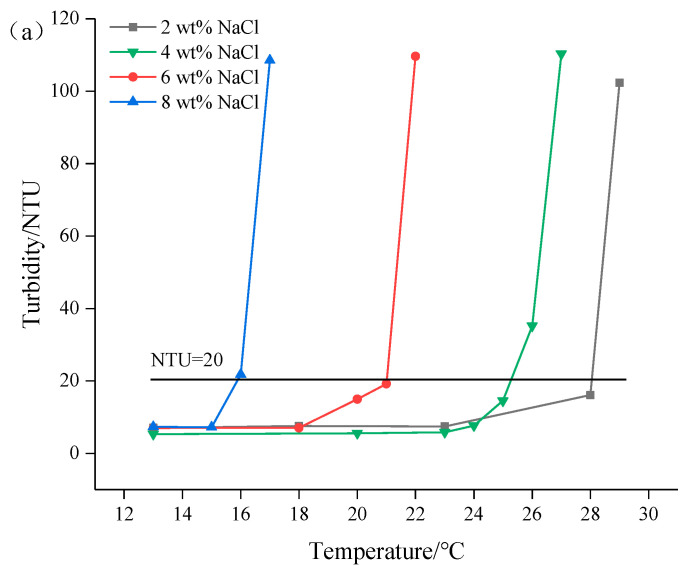

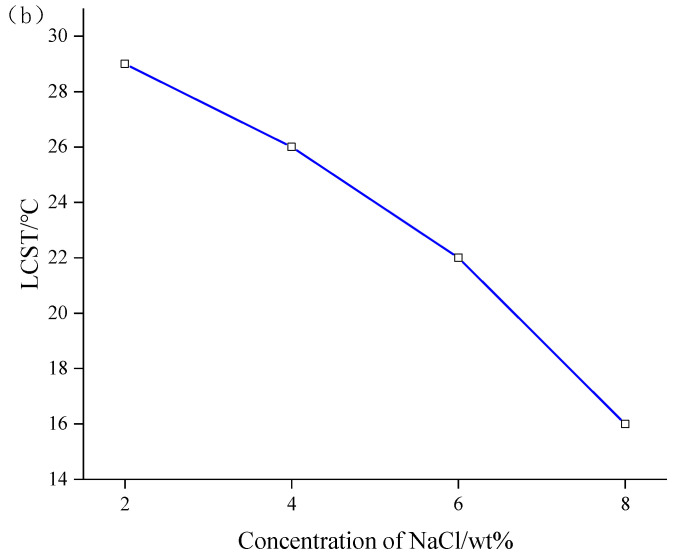
Effects of the NaCl concentration (wt%) on turbidity change with temperature (**a**) and LCST (**b**) of PNBAM aqueous solution.

Figure 9 shows the variation of LCST with NaCl concentration of PNBAM. As can be seen from Figure 9, the LCST of PNBAM reaches 26 °C (4 wt% NaCl) and 16 °C (8 wt% NaCl). As the NaCl concentration continues to rise, the LCST of PNBAM decreases. This is due to the addition of NaCl, which enhances the polarity of water [44,45,46]. The hydration of NaCl further weakens the hydrogen bond between the amide group and water, breaking the hydration layer on the surface of the polymer molecules [44,45,46]. This leads to an enhancement of the intermolecular association of PNBAM, making it more susceptible to temperature, leading to a decrease in the LCST of PNBAM.

#### 2.3.2. Particle Size Analysis

**Figure 10:**
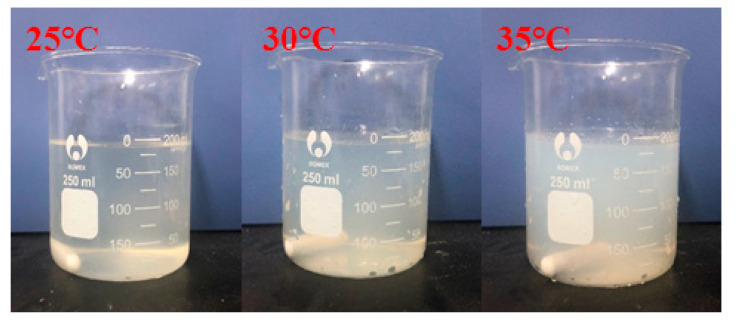
PNBAM aqueous solution graphs at 25, 30, and 35 °C.

Table 4 shows the variation in the particle-size distribution of PNBAM aqueous solutions with temperature. Figure 10 shows the PNBAM aqueous solution graphs at 25, 30, and 35 °C. As can be seen from Table 4 and Figure 10, the introduction of the hydrophobic monomer BA in PNBAM resulted in a hydrophobic association effect before its LCST [22,42,47]. Particle size values are still measurable in aqueous solutions at 25 °C, slightly turbid solutions, and a particle size of 337.4 nm. With the increase in temperature, the LCST of PNBAM was reached, with a particle size of 593.9 nm at 30 °C. With a further increase in temperature, the PNBAM solution became more turbid at 35 °C, with a particle size of 1241 nm, indicating a progressively more intense temperature-sensitive effect.

The number of size peaks found and the variation of the peaks show that two small size peaks appear at 25 °C. At this point, only the hydrophobic chain segment from the hydrophobic monomer BA acts on the PNBAM molecular chain. As the temperature increases, the temperature-sensitive effect gradually increases, with a third particle size peak appearing at 30 °C. This indicates that the temperature-sensitive effect starts to appear at this point. At 35 °C, the temperature-sensitive effect becomes more intense. The hydrophobic properties of the temperature-sensitive side chains increase, with higher peaks and more widely distributed particle size peaks [22,42,47].

#### 2.3.3. Zeta Potential Analysis

**Figure 11:**
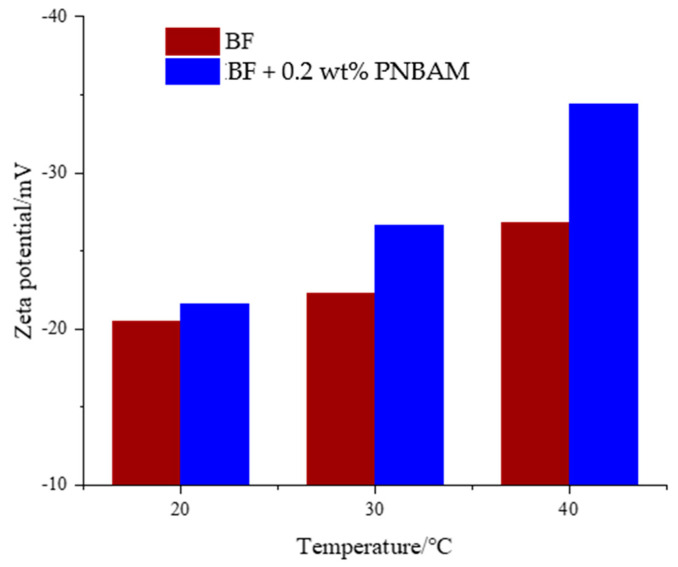
Value of the zeta potential for samples at different temperatures.

Figure 11 shows the variation of the zeta potential of PNBAM with temperature. As can be seen from Figure 11, the absolute value of the negative zeta potential increases slightly with increasing temperature (20 to 30, and 40 °C) for BF without the addition of PNBAM. The absolute value of the negative zeta potential increased from −20.5 to −22.3 to −26.8 mV; however, the increase was not significant. However, for the BF containing 0.2 wt% PNBAM, the absolute value of the negative zeta potential increased continuously with increasing temperature. The absolute value of the negative zeta potential increased significantly from −21.6 to −26.6 to −34.4 mV. In particular, the absolute degree of elevation of the negative zeta potential increased significantly after reaching the LCST of PNBAM.

The effect of PNBAM on the stable structure of the suspension of BF was investigated using zeta potential testing. As can be seen from the graph, the absolute value of the negative zeta potential decreased with increasing temperature before and after the LCST of PNBAM, regardless of whether the BF contained PNBAM or not, from 20 to 40 °C. This indicates that the dispersibility of the bentonite became weaker at low temperatures. The addition of PBANM increased the absolute value of zeta potential compared to BF. This is due to the adsorption of PNBAM on the clay surface increasing the thickness of the hydration film of the clay particles [37,39,43].

#### 2.3.4. Mechanism Analysis

**Figure 12:**
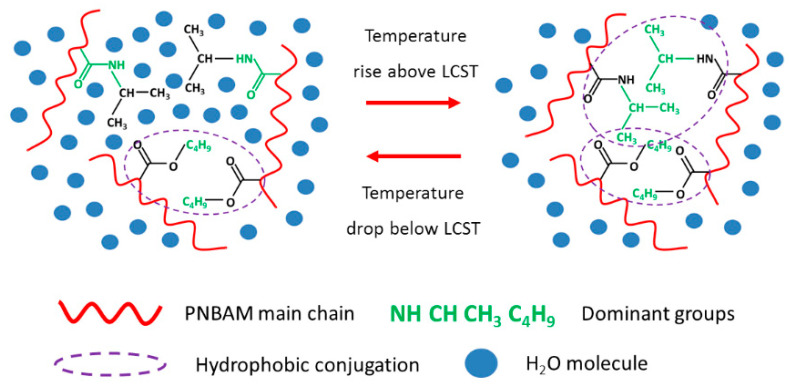
Rheology modulation process of PNBAM at different temperatures.

Figure 12 shows the rheology modulation process of PNBAM at different temperatures. As can be seen in Figure 12, PNBAM molecules have specific temperature-sensitive side chains. The temperature-sensitive side chains consist of isopropyl and amide groups. When the temperature is below the LCST of PNBAM, water is highly polarized, and the hydrogen bonding between the amide group and water molecules is strong. The temperature-sensitive side chains are hydrophilic. The hydrophobic linkages between the PNBAM molecular chains are weak and have a weak effect on the rheology of the drilling fluid. The measured particle size distribution and absolute zeta potential values are lower at this point.

The polarity of water becomes weaker at higher temperatures, and the hydrogen bonding between the amide groups on the temperature-sensitive side chains and the water molecules becomes weaker. At temperatures above LCST, the hydrophobic group (isopropyl) dominates, and the temperature-sensitive side chains become hydrophobic. The hydrophobic bonds between the PNBAM molecular chains increase, resulting in a stronger 3D network structure between the polymer molecular chains and improved strength of the internal spatial structure of the drilling fluid. The measured particle size distribution and absolute values of the zeta potential are higher at this point [44,45,46].

External factors mostly affect the LCST of PNBAM by influencing the strength of hydrogen bonding between the temperature-sensitive side chains of PNBAM and water molecules. An excessive concentration of PNBAM brings about a higher number of hydrogen bonds, which to some extent improves the LCST of PNBAM; however, the effect is not significant. In contrast, salt further weakens the hydrogen bonding between the amide group and water by enhancing the polarity of water and its hydration, destroying the hydration layer on the surface of the polymer molecule and substantially reducing the LCST of PNBAM [44,45,46].

In previous studies, conventional rheology modifiers have been used to achieve thickening through the properties of their own groups (adsorption, cross-linking, etc.) and the spreading of polymer molecular chains. These approaches, however, do not respond to specific temperature bands and have the disadvantage of becoming less effective with increasing temperature. The rheology modifier PNBAM in this study, however, is able to respond effectively to a specific temperature range, with a significant and dramatic response. The thermal behavior of PNBAM is also characterized by a gradual increase with temperature, which effectively compensates for the rheological decline of BF with increasing temperature [32,43].

## 3. Conclusions

In this paper, PNBAM, a temperature-sensitive polymeric rheology modifier for deepwater water-based drilling fluids of flat rheology, was synthesized using emulsion polymerization. The LCST of 0.2 wt% PNBAM in water was 31 °C. PNBAM was more effective than XC in low-temperature rheological regulation of BF. PNBAM was able to resist high temperatures of 150 °C and 30 wt% NaCl and 1.0 wt% CaCl_2_. WBDF-2 with PNBAM demonstrated good rheological and filtration performance and excellent performance of flat rheology from 4 to 65 °C: AV_4°C_:AV_25°C_ < 1.30 and PV_4°C_:PV_25°C_ < 1.20.

The increase in temperature weakens the polarity of water and weakens the hydrogen bonding force between the amide group and water on the temperature-sensitive side chain of PNBAM. This results in a hydrophilic to hydrophobic change of the tempera-ture-sensitive side chains of PNBAM. At this time, the hydrophobic forces between PNBAM molecules increase, and hydrophobic bonding is stronger. This situation leads to stronger intermolecular self-assembly and the formation of 3D network hydrophobic domains. Based on this enhanced temperature-sensitive effect with increasing temperature, PNBAM helps to assist in the level rheology of water-based drilling fluids.

PNBAM still has certain limitations. The resistance to temperature can only reach 150 °C, which makes it difficult to match the higher formation temperature requirements. Due to the introduction of hydrophobic monomers, there is a certain side effect of low-temperature thickening. There is also a certain rheological drop in BF at higher temperatures (120 °C or even higher). This is also difficult to meet with PNBAM. Future research is still needed to develop rheological modifiers adapted to the rheological changes in BF at this temperature range. Of course, avoiding the side effects of the introduction of hydrophobic monomers is also a focus of subsequent research.

## 4. Materials and Methods

### 4.1. Materials

*N*-isopropyl acrylamide (NIPAM, 99 wt% with p-hydroxyanisole MEHQ stabilizer), acrylamide (AM, 99 wt%), butyl acrylate (BA, 99 wt%), azobisisobutyronitrile (AIBN, 98 wt%), sodium chloride (NaCl, 99.5 wt%), calcium chloride dihydrate (CaCl_2_, 99 wt%), Anhydrous sodium carbonate (Na_2_CO_3_, 99.5 wt%) xanthan gum (XC, 99 wt%) and Alkaline alumina (75 wt%) were obtained from Shanghai Aladdin biochemical Technology Co., Ltd. In Shanghai, China. Alkylphenol ether juanyl succinate sodium salt (MS-1, 40 wt%) was obtained from Hubei Kovod Chemical Co., Ltd. In Hubei, China. Bentonite (Industrial grade), was obtained from China Huaian Group., Ltd. In Hubei, China. Low-viscosity polyanionic cellulose (PAC-LV, 95 wt%), amino polyol shale-inhibitive agent (AP-1, 95 wt%), methyl oleate (95 wt%), and barite (industrial grade) were obtained from Shandong Deshunyuan Petroleum Technology Co., Ltd. in Shandong, China.

### 4.2. Experimental Methods

#### 4.2.1. Synthesis of Rheology Modifier PNBAM

Based on the requirements for the evaluation of the flat rheology performance of deepwater water-based drilling fluids [21,22], NIPAM with an LCST of 32–35 °C for the self-polymerization product was chosen as the main body of the synthesis. AM with a similar structure, strong hydrophilicity, and easy participation in the reaction was selected as the hydrophilic monomer to improve the tackifying effect of the product. Butyl acrylate, which is hydrophobic and can easily participate in the reaction, was chosen as the hydrophobic monomer to reduce the LCST of the product due to the introduction of the hydrophilic monomer AM.

The rheology modifier PNBAM was synthesized by using free-radical emulsion polymerization. First, the solution was obtained from 10 g NIPAM dissolved in 80 mL water. The solution was filtered after adding alkaline alumina to remove the aggregation inhibitor p-hydroxyanisole (MEHQ). Then, the mixed solution to be used was obtained with 1.06 g AM, and 0.96 g BA was added to the solution before stirring (molar ratio NIPAM:AM:BA = 12:2:1).

**Figure 13:**
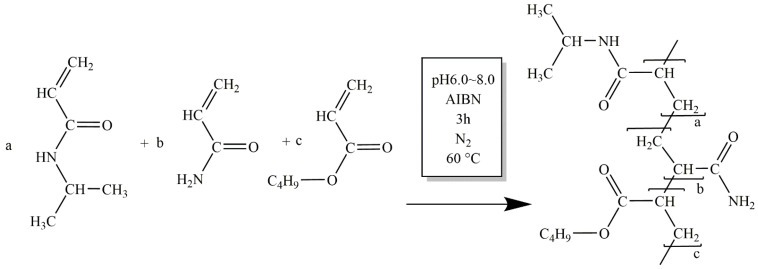
Chemical structure of PNBAM.

The mixed solution and 120 mL of 5# white oil were transferred to a four-necked reaction flask with N_2_ circulation and a mixer. Then, emulsifier MS-1 1 g was added. The mixed solution was heated to 60 °C and stabilized, and then the initiator AIBN 0.1 g was added. The reaction was kept at a constant temperature of 60 °C and stirred for 3 h. The mixture was washed successively with acetone and ethanol. Finally, the precipitate was dried and crushed to obtain rheology modifier P(NIPAM-AM-BA), hereinafter referred to as PNBAM (Figure 13).

#### 4.2.2. Characterization

1. FT-IR

The infrared spectrum of PNBAM was tested using infrared spectroscopy (IRTRacer-1000, Shimadzu, Kyoto, Japan). The PNBAM sample was dried in an oven at 105 °C for 24 h. The sample was mixed with KBr and ground using an agate mortar. The FT-IR spectra of PNBAM were measured at room temperature using the KBr compression method (2 MPa, 3 min) in the wavenumber range of 400–4000 cm^−1^ with a resolution of 4 cm^−1^.

2. TGA

The thermo-gravimetric analysis of PNBAM was tested by using thermogravimetric analysis (TGA2, Mettler, New York, NY, USA). PNBAM was placed in an oven at 105 °C for 24 h and then placed in an equilibrated crucible. The test was started according to the instrument program, with a set temperature range of 30–800 °C, a temperature rise rate of 10 °C/min, and a protective gas of N_2_. A thermogravimetric analysis graph of PNBAM was obtained.

3. ^1^H-NMR spectra

The nuclear magnetic resonance (NMR) spectra of PNBAM were tested by nuclear magnetic resonance spectroscopy (Bruker Analytik, Rheinstetten, Germany) using D_2_O as solvent. For the test, approximately 3 mg of sample was dissolved in heavy water (TMS internal standard, 0.03% volume fraction), and the signal was collected in 16 cycles.

#### 4.2.3. Performance Evaluation

In this study, 4 wt% sodium bentonite suspension was used as the base fluid (BF) for testing because a majority of water-based drilling fluids (WBDF) are prepared using 2–4 wt% bentonite to tackifying and reduce the filtration at low costs [33,36,48].

Configuration of one 4 wt% sodium bentonite suspension: Add 400 mL of distilled water to a high stirring cup, then add 16 g of bentonite and 1.2 g of Na_2_CO_3_ to the distilled water and stir at high speed (6000 r/min) for 20 min, stopping twice to scrape off the clay adhering to the walls of the cup, and leave in a closed container for 24 h [33,36,48]. A 4 wt% sodium bentonite suspension is obtained, and the performance of the 4 wt% sodium bentonite suspension must comply with the standard GBT 16783.1-2014. The composition of one BF can be seen in Table 5.

1. Low temperature rheological performance tests

The rheological performance was tested by using a viscometer (ZNN-D6B, Tongchun Petroleum Instrument, Qingdao, China). The mass fraction at 0.2 wt% of PNBAM and mass fraction at 0.2 wt% of XC were added to the BF and then aged at 150 °C for 16 h using a high temperature roller heating oven. After ageing, a constant temperature and humidity chamber were used to control the temperature, and a ZNN-D6B viscometer was used to test the parameters of BF (θ600, θ300, θ6, θ3) at 4 °C, 15 °C, 25 °C, 35 °C, 45 °C, and 65 °C. The apparent viscosity (AV), plastic viscosity (PV), and yield value (YP) of BF at 4 °C, 15 °C, 25 °C, 35 °C, 45 °C, and 65 °C were also calculated according to the API standard. The parameter ratios of AV, PV, and YP at 4 °C to 25 °C and 4 °C to 65 °C were also compared.

AV = 0·θ600(1)

PV = θ600 − θ300(2)

YP = 0.5·(θ300 − PV)(3)

where AV is the apparent viscosity (mPa·s), PV is the plastic viscosity (mPa·s), and YP is the yield value (Pa).

2. Temperature resistance tests

The BF containing 0.2 wt% of PNBAM was subjected to high temperature hot rolling ageing tests at different temperatures [36,48]. The hot rolling ageing time was 16 h, and the ageing temperatures were 90, 120, 150, and 180 °C. The AV, PV, YP, and API Filtration (FL_API_) of BF after hot rolling ageing were measured by using a ZNN-D6B viscometer according to API standards. The performance of BF before and after hot rolling ageing was compared to evaluate its temperature resistance. To evaluate the temperature resistance of PNBAM, the performance before and after hot rolling ageing with BF was compared.

3. Salt and calcium resistance tests

The BF containing 0.2 wt% of PNBAM was subjected to salt and calcium resistance experiments [36,48]. The mass fractions at 10, 20, and 30 wt% of NaCl were used for the salt resistance experiments. The mass fractions at 0.5 and 1.0 wt% of CaCl_2_ were used for the calcium resistance experiments. To evaluate the salt and calcium resistance of PNBAM, the BF was subjected to high temperature hot roll ageing tests (120 °C and 16 h), and the AV, PV, and YP of the aged BF were tested according to API standards after ageing tests.

4. Drilling fluid system tests

The deepwater water-based drilling fluid system (WBDF) was constructed with the developed rheology modifier PNBAM as the core, under the premise of preferring other drilling fluid additives. The preferred drilling fluid system is as Table 6.

#### 4.2.4. Mechanism Analysis

1. LCST

Determination of LCST is the temperature at which the aqueous solution of the polymer changes from dissolved to insoluble or turbid when the temperature is increased [22,42,47]. Variations in the LCST of polymers exist due to the conditions of their synthesis and external factors. Utilizing the change in turbidity of the solution when the temperature-sensitive effect of the polymer occurs. The LCST was determined by measuring the change in turbidity of the PNBAM solution using a turbidimeter (WZG-200, Shanke apparatus Co., Shanghai, China). Based on the definition of turbidity, a turbidity increase of 20 NTU for PNBAM solutions was chosen as the limit of turbidity at which temperature-sensitive effects occur.

2. Effect of PNBAM concentration on LCST

To study the effect of the PNBAM concentration on the LCST, PNBAM solutions with the mass fraction of 0.05, 0.10, 0.15, 0.20, and 0.25 wt% were prepared, and the change in turbidity with temperature was measured using a WZG-200 turbidimeter to determine its LCST.

3. Effect of salt concentration on LCST

To study the effect of salt concentration on the LCST of PNBAM. The mass fraction at 0.2 wt% of PNBAM was prepared with the salt mass fraction of 2, 4, 6, and 8 wt%, and the LCST was determined by measuring the change in turbidity of the solution with temperature using the turbidimeter.

4. Particle size

The particle size distribution of PNBAM solutions with a mass fraction of 0.2 wt% at different temperatures (20, 30, and 40 °C) was analyzed by using a nano Zetasizer (ZS90, Marven, Malvern City, UK).

5. Zeta potential

The stability of bentonite suspensions can be studied by measuring the zeta potential of the bentonite. The zeta potential of BF containing 0–0.2 wt% PNBAM at different temperatures (20, 30, and 40 °C) was measured with a Zeta Potential Analyzer (Nano ZS, Malvern, Malvern City, UK).

## Figures and Tables

**Table 1:** Parameter ratios of different BFs.

Parameter Ratios	BF	BF + 0.2 wt% XC	BF + 0.2 wt% PNABAM
AV_4°C_:AV2_5°C_	2.23	1.72	1.35
AV_4°C_:AV_65°C_	4.75	3.31	2.25
PV_4°C_:PV_25°C_	2.14	1.77	1.22
PV_4°C_:PV_65°C_	5.00	3.20	2.44
YP_4°C_:YP_25°C_	2.67	1.57	2.5
YP_4°C_:YP_65°C_	4.00	3.67	1.67

**Table 2:** General properties of drilling fluid systems.

Conditions	Density/g/cm^3^	AV/mPa·s	PV/mPa·s	YP/Pa	G′/G″Pa	FL_API_/mL	FL_HTHP_/mL
Before ageing	1.5	45	36	9	8/11	4	-
After 120 °C/16 h ageing	1.5	62	44	18	3/8	4.4	10.4

**Table 3:** Parameter ratios for different drilling fluid systems.

Parameter Ratios	WBDF-1	WBDF-2
AV_4°C_:AV2_5°C_	1.36	1.27
AV_4°C_:AV_65°C_	1.93	1.81
PV_4°C_:PV_25°C_	1.40	1.19
PV_4°C_:PV_65°C_	1.92	1.68
YP_4°C_:YP_25°C_	1.14	1.42
YP_4°C_:YP_65°C_	2.00	2.06

**Table 4:** Variation in the particle-size distribution of PNBAM aqueous solutions with temperature.

Temperature/°C	Particle Size	Particle Size Distribution Graph
25	D_10_ = 177 nm D_50_ = 255 nm D_ave_ = 337.4 nm	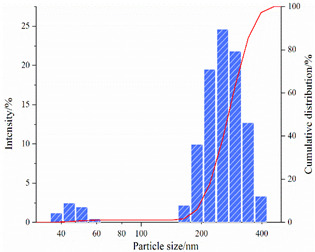
30	D_10_ = 205 nm D_50_ = 648 nm D_ave_ = 593.8 nm	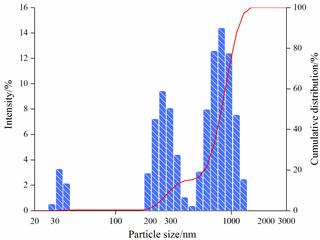
35	D_10_ = 1050 nm D_50_ = 1330 nm D_ave_ = 1241 nm	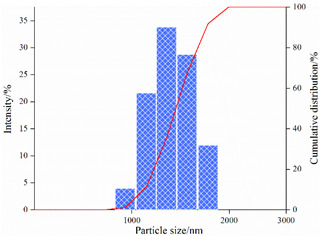

**Table 5:** Composition of one BF.

Component	Additions
water	400 mL
bentonite	16 g
Na_2_CO_3_	1.2 g

**Table 6:** Composition of the WBDFs.

Component	Function	Mass Fraction/wt%	
		WBDF-1	WBDF-2
bentonite	filtrate reducer and viscosifier	4	4
PNBAM	rheology modifier	0	0.2
XC	viscosifier	0.2	0
PAC-LV	filtrate reducer	1.5	1.5
AP-1	shale inhibitor	1	1
Methyl oleate	lubricants	2	2
NaCl	hydrate inhibitor	20	20

Note: drilling fluid systems were weighted to 1.5 g/cm^3^ by barite.
